# Supplementary material for: Bone marrow stromal cells induce chromatin remodeling in multiple myeloma cells leading to transcriptional changes
Source: Nat Commun. 2024 May 16;15:4139. doi: 10.1038/s41467-024-47793-5 (PMC11098817; doi:10.1038/s41467-024-47793-5)
Supplement: Supplementary file 1 — Supplementary Information [file 41467_2024_47793_MOESM1_ESM.pdf]

SUPPLEMENTARY FIGURE 1

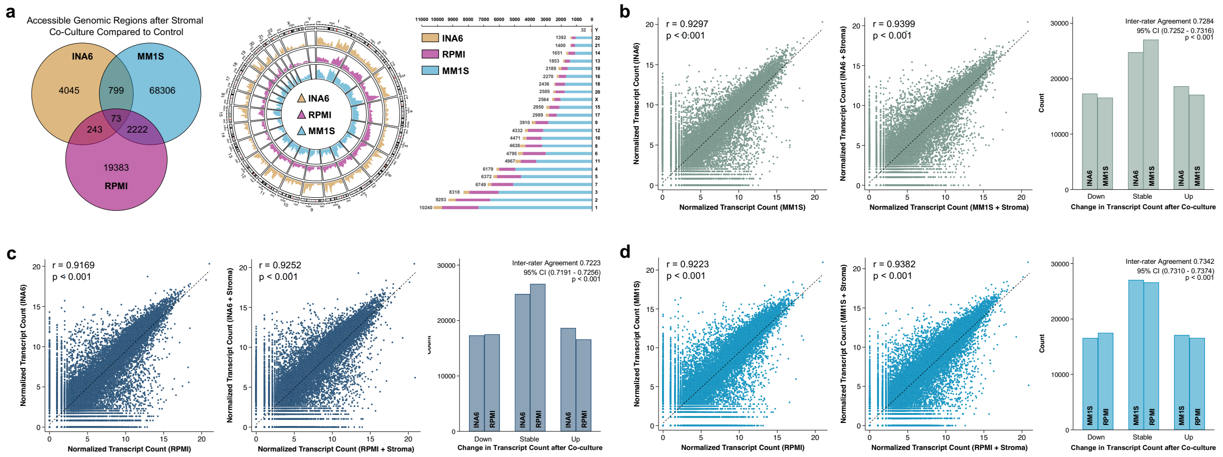

Supplementary Figure 1 Gene expression and chromatin accessibility of individual myeloma cell lines in response to stromal co-culture. **a**, Euler diagram (left panel), circular area plots (middle panel), and bar graphs (right panel) showing the localization and overlap of the genomic regions with gained accessibility after stromal co-culture stratified by myeloma cell line. **b**, Scatter plots showing the pairwise correlation between INA6 and MM1S gene expression at baseline (left panel) and after stromal co-culture (middle panel). Bar graphs showing the agreement of direction in gene expression between INA6 and MM1S after stromal co-culture (right panel). **c**, Scatter plots showing the pairwise correlation between INA6 and RPMI gene expression at baseline (left panel) and after stromal co-culture (middle panel). Bar graphs showing the agreement of direction in gene expression between INA6 and RPMI after stromal co-culture (right panel). **d**, Scatter plots showing the pairwise correlation between MM1S and RPMI gene expression at baseline (left panel) and after stromal co-culture (middle panel). Bar graphs showing the agreement of direction in gene expression between MM1S and RPMI after stromal co-culture (right panel). The pairwise correlation coefficients in 1b-d represent Pearson correlation coefficients for all transcripts (n = 60675).

SUPPLEMENTARY FIGURE 2

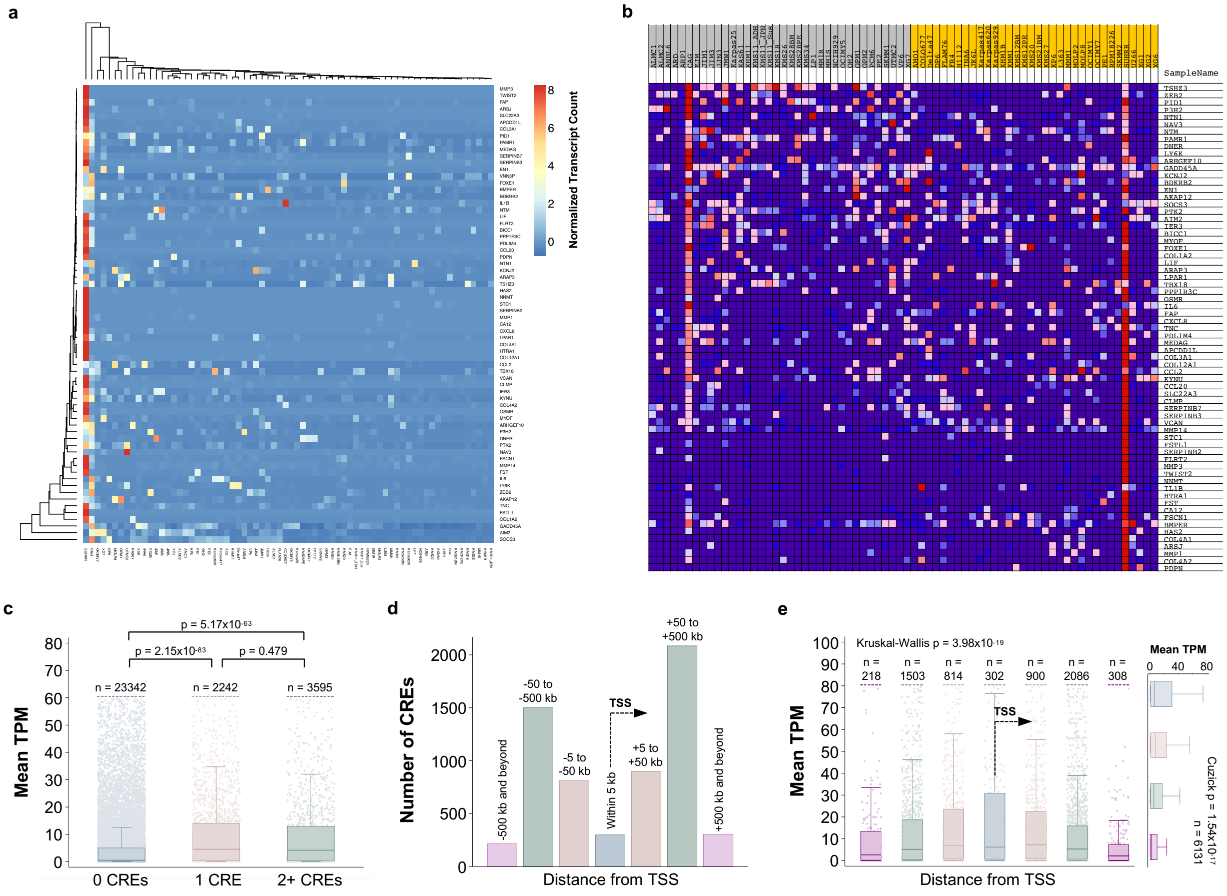

Supplementary Figure 2 Baseline myeloma cell line gene expression and additional analyses of enhancer effects on the expression of the predicted target genes. **a**, Heatmap showing the baseline gene expression of the 68 stroma-induced genes in 70 myeloma cell lines. **b**, Gene set enrichment analysis of the 68 stroma-induced genes stratified by the presence (gray) and absence (yellow) of high-risk IGH translocations among the 70 myeloma cell lines (Enrichment Score -0.57, p = 0.644). High-risk IGH translocations were defined as the presence of t(4;14), t(14;16), or t(14;20). **c**, Box and strip plots demonstrating the association between the presence of CREs with altered chromatin accessibility after stromal co-culture and increased expression of their predicted target genes (Mann-Whitney-U test). There was no dosage effect (i.e. the presence of more than one CRE was not associated with a further increase in gene expression). **d**, Bar graphs showing the distribution of associated CREs with the vast majority being located outside the promoter regions of the predicted target genes. **e**, Box and strip plots showing the expression of the predicted target genes stratified by location and direction of the associated CRE (upstream *versus* downstream and by distance). There was a decrease in the expression of the predicted target genes as distance increased (Kruskal-Wallis test). Data are presented as standard Tukey boxplots (with the box encompassing Q1 to Q3, the median denoted as a central horizontal line in the box, and the whiskers covering the data within ±1.5 IQR in 2e).

SUPPLEMENTARY FIGURE 3

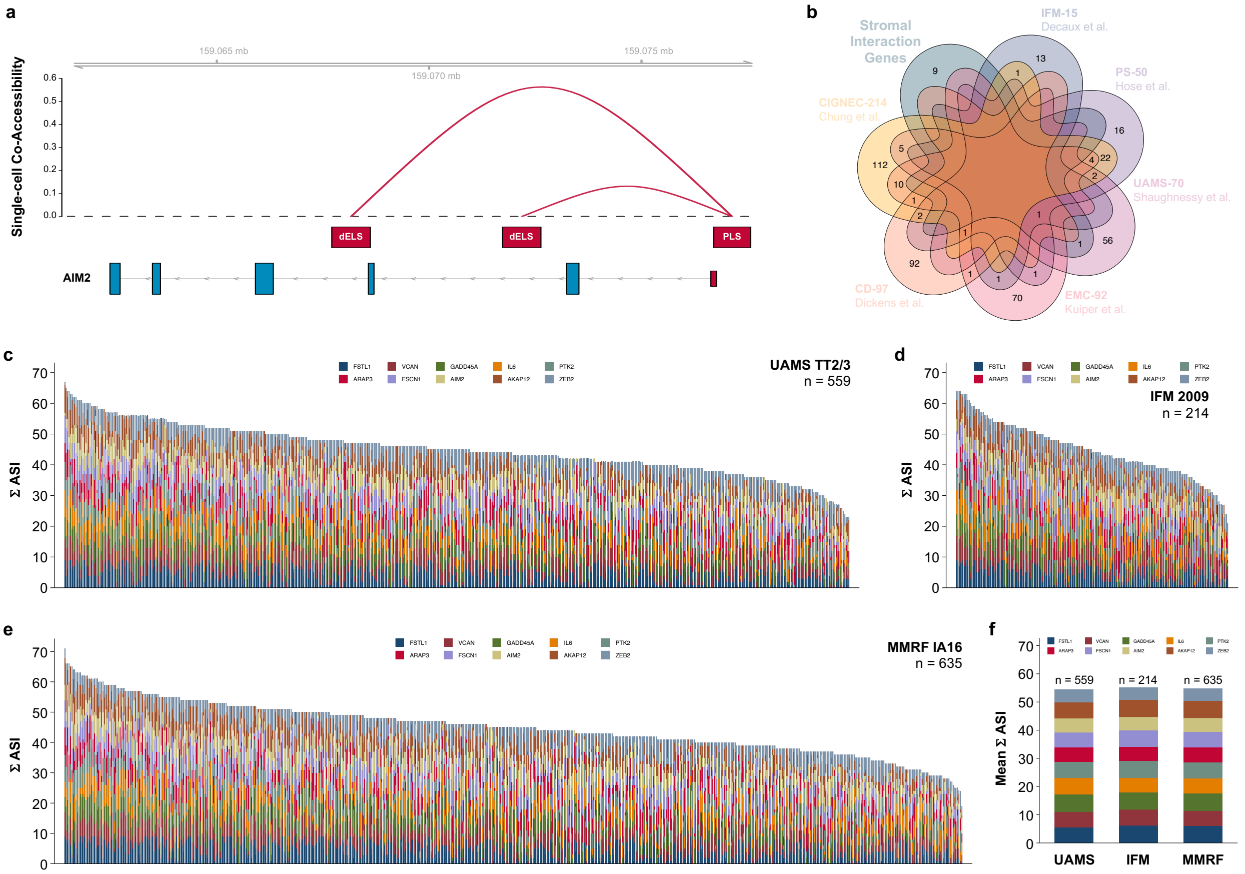

Supplementary Figure 3 Single-cell co-accessibility studies and the contribution of each of the 10 prognostically significant stromal interaction genes to the summary measure Σ ASI. **a**, Connection plot showing the positive single-cell co-accessibility between the *AIM2* promoter region and two intronic distal enhancers predicted to govern *AIM2* expression. **b**, Venn diagram showing the limited, one-gene, overlap of the 10 selected stromal interaction genes with the established high-risk gene expression classifiers in myeloma. **c**, Bar graphs demonstrating the absolute contribution of each individual gene to the Σ ASI score in the derivation cohort (n = 559). **d**, Bar graphs demonstrating the absolute contribution of each individual gene to the Σ ASI score in the first validation cohort (n = 214). **e**, Bar graphs demonstrating the absolute contribution of each individual gene to the Σ ASI score in the second validation cohort (n = 635). **f**, Bar graphs showing the mean contribution of each individual gene to the Σ ASI score among patients considered ASI+ (Σ ASI ≥ 50) in the three patient populations (n = 1408).

REGRESSION MODELS

Gene expression data and progression-free survival outcomes from UAMS TT2/3 (GSE24080) were used for feature selection by employing automated model selection procedures (forward and backward selection with a p-value threshold of 0.200). Forward and backward selection included 13 (**Supplementary Table 1**) and 17 (**Supplementary Table 2**) genes associated with progression-free survival, respectively. The 10 genes selected by both methods (**Supplementary Table 3**) were selected for further study and were used to calculate the 10-gene classifier (**Supplementary Table 4**).

Supplementary Table 1 Multivariable-adjusted proportional hazards regression for progression-free survival (forward selection) in UAMS TT2/3 (GSE24080, n = 559)

Gene	HR (95% CI)	p-value
GADD45A	0.82 (0.71 - 0.94)	0.005
MMP14	0.52 (0.24 - 1.10)	0.085
AIM2	1.33 (1.12 - 1.58)	0.001
FSCN1	0.67 (0.42 - 1.07)	0.093
IL1B	2.12 (1.08 - 4.16)	0.030
FSTL1	0.49 (0.28 - 0.85)	0.011
AKAP12	0.66 (0.46 - 0.93)	0.018
VCAN	0.64 (0.42 - 0.97)	0.034
IL6	1.21 (1.01 - 1.46)	0.043
PTK2	1.28 (0.95 - 1.73)	0.109
FLRT2	0.28 (0.07 - 1.08)	0.065
ARAP3	1.97 (0.90 - 4.30)	0.088
ZEB2	2.89 (0.65 - 12.83)	0.163

HR = Hazard Ratio | CI = Confidence Interval

Supplementary Table 2 Multivariable-adjusted proportional hazards regression for progression-free survival (backward selection) in UAMS TT2/3 (GSE24080, n = 559)

**Gene**	**HR (95% CI)**	**p-value**
AIM2	1.37 (1.16 - 1.62)	< 0.001
BICC1	2.07 (1.19 - 3.61)	0.010
FSTL1	0.45 (0.25 - 0.80)	0.006
PTK2	1.24 (0.91 - 1.68)	0.168
TBX18	0.19 (0.02 - 2.12)	0.178
AKAP12	0.65 (0.46 - 0.93)	0.017
ARAP3	1.72 (0.76 - 3.86)	0.190
FSCN1	0.68 (0.41 - 1.12)	0.131
HAS2	0.63 (0.39 - 1.02)	0.058
VCAN	0.65 (0.43 - 0.99)	0.043
VNN3	0.50 (0.18 - 1.44)	0.200
GADD45A	0.81 (0.70 - 0.93)	0.003
MMP3	0.37 (0.11 - 1.25)	0.109
IL6	1.22 (1.01 - 1.46)	0.038
ZEB2	3.48 (0.76 - 15.91)	0.108
HTRA1	1.53 (0.89 - 2.61)	0.121
FOXE1	1.81 (0.80 - 4.11)	0.154

HR = Hazard Ratio | CI = Confidence Interval

Supplementary Table 3 Multivariable-adjusted proportional hazards regression for progression-free survival in UAMS TT2/3 (GSE24080, n = 559) using the 10 genes included by both forward and backward selection

**Gene**	**HR (95% CI)**	**p-value**
GADD45A	0.80 (0.70 - 0.91)	0.001
AIM2	1.29 (1.10 - 1.51)	0.002
FSCN1	0.50 (0.32 - 0.78)	0.002
FSTL1	0.46 (0.27 - 0.77)	0.003
AKAP12	0.68 (0.48 - 0.96)	0.029
VCAN	0.69 (0.46 - 1.01)	0.058
IL6	1.20 (1.00 - 1.45)	0.051
PTK2	1.29 (0.97 - 1.71)	0.081
ARAP3	1.60 (0.81 - 3.15)	0.173
ZEB2	5.09 (1.29 - 20.14)	0.020

HR = Hazard Ratio | CI = Confidence Interval

Supplementary Table 4 Univariable proportional hazards regression for progression-free survival in UAMS TT2/3 (GSE24080, n = 559) using the 10-gene classifier (score) resulting from the genes included by both forward and backward selection

Gene	HR (95% CI)	p-value
Score	1.06 (1.04 - 1.08)	< 0.001

HR = Hazard Ratio | CI = Confidence Interval

Since progression-free survival outcomes were used for the derivation of the 10-gene classifier, the independent prognostic significance of the dichotomized 10-gene classifier for overall survival was first confirmed in the derivation patient population (**Supplementary Table 5**). The dichotomized 10-gene classifier was then validated in two independent patient populations (**Supplementary Table 6**, **Supplementary Table 7**). To increase power, the two validation patient populations were pooled to test the independence of the dichotomized 10-gene classifier of the UAMS-70 and EMC-92 classifier (**Supplementary Table 8**, **Supplementary Table 9**).

Supplementary Table 5 Multivariable-adjusted proportional hazards regression for overall survival in UAMS TT2/3 (GSE24080, n = 559) using the dichotomized 10-gene classifier (ASI+)

Gene	HR (95% CI)	p-value
Age	1.02 (1.00 - 1.03)	0.044
Sex	1.11 (0.81 - 1.51)	0.512
ISS	1.69 (1.36 - 2.11)	< 0.001
LDH	2.40 (1.71 - 3.37)	< 0.001
ASI+	1.79 (1.32 - 2.42)	< 0.001

HR = Hazard Ratio | CI = Confidence Interval | ISS = International Staging System | LDH = Elevated Lactate Dehydrogenase | No evidence for violations of the proportional hazards assumption (p = 0.294)

Supplementary Table 6 Multivariable-adjusted proportional hazards regression for overall survival in IFM 2009 (n = 152) using the dichotomized 10-gene classifier (ASI+)

Gene	HR (95% CI)	p-value
Age	1.00 (0.96 - 1.04)	0.901
Sex	0.84 (0.48 - 1.47)	0.539
ISS	1.26 (0.85 - 1.86)	0.246
HRT	3.01 (1.43 - 6.32)	0.004
Del(17p)	2.79 (1.41 - 5.50)	0.003
Gain(1q)	2.23 (1.25 - 3.99)	0.007
Del(1p)	0.89 (0.49 - 1.62)	0.702
ASI+	2.04 (1.16 - 3.60)	0.013

HR = Hazard Ratio | CI = Confidence Interval | ISS = International Staging System | HRT = High-risk IGH Translocation | No evidence for violations of the proportional hazards assumption (p = 0.228)

Supplementary Table 7 Multivariable-adjusted proportional hazards regression for overall survival in MMRF IA16 (n = 447) using the dichotomized 10-gene classifier (ASI+)

Gene	HR (95% CI)	p-value
Age	1.04 (1.02 - 1.06)	< 0.001
Sex	1.47 (1.04 - 2.08)	0.028
ISS	1.47 (1.18 - 1.83)	0.001
LDH	1.92 (1.26 - 2.92)	0.002
HRT	2.01 (1.32 - 3.08)	0.001
Del(17p)	1.42 (0.88 - 2.28)	0.154
Gain(1q)	1.51 (1.08 - 2.11)	0.017
ASI+	1.55 (1.11 - 2.17)	0.010

HR = Hazard Ratio | CI = Confidence Interval | ISS = International Staging System | LDH = Elevated Lactate Dehydrogenase | HRT = High-risk IGH Translocation | No evidence for violations of the proportional hazards assumption (p = 0.225)

Supplementary Table 8 Multivariable-adjusted proportional hazards regression for overall survival in IFM 2009 and MMRF IA16 (n = 723) using the dichotomized 10-gene classifier (ASI+)

Gene	HR (95% CI)	p-value
Population	0.74 (0.41 - 1.31)	0.299
Age	1.03 (1.02 - 1.05)	< 0.001
Sex	1.45 (1.12 - 1.88)	0.005
ISS	1.50 (1.26 - 1.77)	< 0.001
HRT	1.60 (1.16 - 2.20)	0.005
del(17p)	2.51 (1.35 - 4.66)	0.004
UAMS-70	2.60 (1.88 - 3.58)	< 0.001
ASI+	1.33 (1.02 - 1.73)	0.038

HR = Hazard Ratio | CI = Confidence Interval | Population = MMRF versus IFM | ISS = International Staging System | HRT = High-risk IGH Translocation | No evidence for violations of the proportional hazards assumption (p = 0.932)

Supplementary Table 9 Multivariable-adjusted proportional hazards regression for overall survival in IFM 2009 and MMRF IA16 (n = 723) using the dichotomized 10-gene classifier (ASI+)

Gene	HR (95% CI)	p-value
Population	0.74 (0.41 - 1.31)	0.300
Age	1.03 (1.02 - 1.05)	< 0.001
Sex	1.39 (1.07 - 1.80)	0.013
ISS	1.54 (1.30 - 1.81)	< 0.001
HRT	1.37 (0.97 - 1.91)	0.071
del(17p)	2.58 (1.39 - 4.77)	0.003
EMC-92	2.73 (1.95 - 3.83)	< 0.001
ASI+	1.38 (1.06 - 1.79)	0.017

HR = Hazard Ratio | CI = Confidence Interval | Population = MMRF versus IFM | ISS = International Staging System | HRT = High-risk IGH Translocation | No evidence for violations of the proportional hazards assumption (p = 0.877)

We were interested in investigating the association between the presence of adverse stromal interactions and the development of therapeutic resistance beyond progression-free survival outcomes. We examined response to therapy with novel agents at 3 months after treatment initiation to remove the potential confounding effect of high-dose chemotherapy (given as conditioning in those patients undergoing autologous hematopoietic stem cell transplantation as part of their first-line treatment). Defining PD/SD by IMWG unified response criteria as "early therapeutic resistance", we tested the hypothesis that the dichotomized 10-gene classifier is associated with this lack of early response to treatment. Considering all first-line regimens, the sample size allowed multivariable-adjusted modeling (**Supplementary Table 10**). Considering only patients receiving first-line VRd, the sample size was too limited for extensive modeling and we therefore fit univariable models for each potential predictor separately (**Supplementary Table 11**).

Supplementary Table 10 Multivariable-adjusted unconditional logistic regression for early therapeutic resistance to any first-line regimen (PD/SD by IMWG unified response criteria at 3 months) in MMRF (n = 396) using the dichotomized 10-gene classifier (ASI+)

Gene	OR (95% CI)	p-value
Age	1.02 (1.00 - 1.05)	0.091
Sex	0.92 (0.52 - 1.62)	0.774
ISS	0.92 (0.64 - 1.33)	0.676
LDH	1.02 (0.43 - 2.40)	0.959
HRT	0.68 (0.26 - 1.75)	0.423
Del(17p)	0.55 (0.20 - 1.53)	0.253
Gain(1q)	1.16 (0.63 - 2.13)	0.633
UAMS-70	0.51 (0.17 - 1.50)	0.221
EMC-92	1.79 (0.63 - 5.08)	0.277
ASI+	2.45 (1.36 - 4.41)	0.003

OR = Odd Ratio | CI = Confidence Interval | ISS = International Staging System | LDH = Elevated Lactate Dehydrogenase | HRT = High-risk Translocation | Good calibration (Hosmer-Lemeshow p = 0.893) and acceptable discrimination (C = 0.646)

Supplementary Table 11 Univariable unconditional logistic regression for early therapeutic resistance to first-line VRd (PD/SD by IMWG unified response criteria at 3 months) in MMRF (n = 211) using the dichotomized 10-gene classifier (ASI+)

Gene	OR (95% CI)	p-value
Age	1.03 (0.98 - 1.07)	0.243
Sex	0.96 (0.42 - 2.18)	0.916
ISS	1.19 (0.69 - 2.06)	0.524
HRT	0.58 (0.13 - 2.65)	0.483
Del(17p)	0.82 (0.18 - 3.84)	0.806
Gain(1q)	1.19 (0.45 - 3.14)	0.723
Del(1p)	0.62 (0.14 - 2.81)	0.536
UAMS-70	0.30 (0.04 - 2.32)	0.247
EMC-92	1.03 (0.98 - 1.07)	0.243
ASI+	2.62 (1.14 - 6.03)	0.023

OR = Odd Ratio | CI = Confidence Interval | ISS = International Staging System | HRT = High-risk Translocation

We then showed that the development of early therapeutic resistance translated into inferior progression-free survival among patients receiving first-line VRd in MMRF IA16 (n = 211) and in IFM 2009 (n = 214). The models below demonstrate that the presence of adverse stromal interactions predict progression-free survival independent of the responder status (depth of response). Since the best response to treatment is unknown at baseline it potentially represents a time-varying covariate, which makes the interpretation of coefficients from standard proportional hazards models (**Supplementary Table 12**, **Supplementary Table 13**) difficult. Instead of engaging in more complicated modeling of time-varying covariates, we show here that the effect of adverse stromal interactions on progression-free survival remains consistent among responders (PR or better). This suggests that the prognostic significance of adverse stromal interactions is not just due to identifying the non-responders (**Supplementary Table 14**, **Supplementary Table 15**).

Supplementary Table 12 Multivariable-adjusted proportional hazards regression for progression-free survival in IFM 2009 (first-line VRd only, n = 214) using the dichotomized 10-gene classifier (ASI+)

Gene	HR (95% CI)	p-value
PD / SD	1.00 (reference)	
PR	0.03 (0.01 - 0.11)	< 0.001
VGPR	0.02 (0.01 - 0.06)	< 0.001
CR / sCR	0.01 (0.00 - 0.02)	< 0.001
ASI+	1.73 (1.25 - 2.40)	0.001

HR = Hazard Ratio | CI = Confidence Interval | PD = Progressive Disease | SD = Stable Disease | PR = Partial Response | VGPR = Very Good Partial Response | CR = Complete Response | sCR = Stringent Complete Response | No evidence for violations of the proportional hazards assumption (p = 0.668)

Supplementary Table 13 Multivariable-adjusted proportional hazards regression for progression-free survival in MMRF IA16 (first-line VRd only, n = 211) using the dichotomized 10-gene classifier (ASI+)

Gene	HR (95% CI)	p-value
PD / SD	1.00 (reference)	
PR	0.29 (0.09 - 1.02)	0.053
VGPR	0.12 (0.03 - 0.38)	< 0.001
CR / sCR	0.01 (0.02 - 0.18)	< 0.001
ASI+	1.66 (1.13 - 2.44)	0.010

HR = Hazard Ratio | CI = Confidence Interval | PD = Progressive Disease | SD = Stable Disease | PR = Partial Response | VGPR = Very Good Partial Response | CR = Complete Response | sCR = Stringent Complete Response | No evidence for violations of the proportional hazards assumption (p = 0.806)

Supplementary Table 14 Univariable proportional hazards regression for progression-free survival in IFM 2009 (first-line VRd only, responders only, n = 208) using the dichotomized 10-gene classifier (ASI+)

Gene	HR (95% CI)	p-value
ASI+	1.56 (1.13 - 2.17)	0.007

HR = Hazard Ratio | CI = Confidence Interval | No evidence for violations of the proportional hazards assumption (p = 0.110)

Supplementary Table 15 Univariable proportional hazards regression for progression-free survival in IFM 2009 (first-line VRd only, responders only, n = 206) using the dichotomized 10-gene classifier (ASI+)

Gene	HR (95% CI)	p-value
ASI+	1.84 (1.25 - 2.71)	0.002

HR = Hazard Ratio | CI = Confidence Interval | No evidence for violations of the proportional hazards assumption (p = 0.287)

To test the hypothesis that the presence of adverse stromal interactions is associated with disease dissemination we used the (1) definite development of new bone lesions or soft tissue plasmacytomas and (2) the definite increase in the size of existing bone lesions or soft tissue plasmacytomas as a composite endpoint. We fit both univariable (**Supplementary Table 16**) and multivariable-adjusted models (**Supplementary Table 17**).

Supplementary Table 16 Univariable proportional hazards regression for the development or progression of bone lesions or soft tissue plasmacytomas (MMRF IA16, n = 622) using the dichotomized 10-gene classifier (ASI+)

Gene	HR (95% CI)	p-value
ASI+	2.24 (1.51 - 3.31)	< 0.001

HR = Hazard Ratio | CI = Confidence Interval | No evidence for violations of the proportional hazards assumption (p = 0.983)

Supplementary Table 17 Multivariable-adjusted proportional hazards regression for the development or progression of bone lesions or soft tissue plasmacytomas (MMRF IA16, n = 442) using the dichotomized 10-gene classifier (ASI+)

Gene	HR (95% CI)	p-value
Age	1.00 (0.98 - 1.03)	0.699
Sex	1.54 (0.93 - 2.55)	0.092
ISS	1.21 (0.89 - 1.65)	0.213
LDH	1.33 (0.67 - 2.63)	0.413
HRT	0.78 (0.35 - 1.74)	0.550
Del(17p)	1.23 (0.58 - 2.58)	0.590
Gain(1q)	2.01 (1.23 - 3.27)	0.005
ASI+	2.18 (1.35 - 3.51)	0.001

HR = Hazard Ratio | CI = Confidence Interval | ISS = International Staging System | HRT = High-risk IGH Translocation | No evidence for violations of the proportional hazards assumption (p = 0.212)
