# Supplementary material for: Bone marrow stromal cells induce chromatin remodeling in multiple myeloma cells leading to transcriptional changes
Source: Nat Commun. 2024 May 16;15:4139. doi: 10.1038/s41467-024-47793-5 (PMC11098817; doi:10.1038/s41467-024-47793-5)
Supplement: Supplementary file 3 — Description of Additional Supplementary Files [file 41467_2024_47793_MOESM3_ESM.pdf]

Supplementary Data 1

1. ATAC-seq DCA: Detailed information on regions with differential chromatin accessibility and associated predicted target genes.

2. ATAC-seq Unique Regions by Cell: Detailed information on regions with differential chromatin accessibility stratified by cell line.

3. ATAC-seq Target Genes by Cell: Detailed information on regions with differential chromatin accessibility and associated predicted target genes stratified by cell line.

Supplementary Data 2

1. RNA-seq DGE: Detailed information on differential gene expression between experimental conditions (stromal co-culture).

2. RNA-seq DGE Annotation: Functional annotation of the genes differentially expressed between experimental conditions (stromal co-culture).

3. RNA-seq Discordant by Cell: Differentially expressed genes with limited agreement between the three cell lines (transcripts with discordant behavior in response to stromal co-culture, transcripts outside Bland-Altman limits of agreement, n = 4083 transcripts, 6.73% of all transcripts).

4. RNA-seq Discordant Annotation: Functional annotation of the differentially expressed genes with limited agreement between the three cell lines.

Supplementary Data 3

1. Target Gene Annotation: Functional annotation of the predicted target genes.

Supplementary Software 1

R and Stata code to calculate the S ASI as a summary measure of adverse stromal interactions as well as example data and documentation
