# Supplementary material for: Bone marrow stromal cells induce chromatin remodeling in multiple myeloma cells leading to transcriptional changes
Source: Nat Commun. 2024 May 16;15:4139. doi: 10.1038/s41467-024-47793-5 (PMC11098817; doi:10.1038/s41467-024-47793-5)
Supplement: Supplementary file 8 — Reporting Summary [file 41467_2024_47793_MOESM8_ESM.pdf]

nature portfolio

Corresponding author(s): Mehmet K. Samur, PhD
Nikhil C. Munshi, MD

Last updated by author(s): Feb 29, 2024

Reporting Summary

Nature Portfolio wishes to improve the reproducibility of the work that we publish. This form provides structure for consistency and transparency in reporting. For further information on Nature Portfolio policies, see our Editorial Policies and the Editorial Policy Checklist.

Statistics

For all statistical analyses, confirm that the following items are present in the figure legend, table legend, main text, or Methods section.

n/a	Confirmed	
☐	☒	The exact sample size (*n*) for each experimental group/condition, given as a discrete number and unit of measurement
☐	☒	A statement on whether measurements were taken from distinct samples or whether the same sample was measured repeatedly
☐	☒	The statistical test(s) used AND whether they are one- or two-sided<br>*Only common tests should be described solely by name; describe more complex techniques in the Methods section.*
☐	☒	A description of all covariates tested
☐	☒	A description of any assumptions or corrections, such as tests of normality and adjustment for multiple comparisons
☐	☒	A full description of the statistical parameters including central tendency (e.g. means) or other basic estimates (e.g. regression coefficient) AND variation (e.g. standard deviation) or associated estimates of uncertainty (e.g. confidence intervals)
☐	☒	For null hypothesis testing, the test statistic (e.g. *F*, *t*, *r*) with confidence intervals, effect sizes, degrees of freedom and *P* value noted<br>*Give P values as exact values whenever suitable.*
☒	☐	For Bayesian analysis, information on the choice of priors and Markov chain Monte Carlo settings
☒	☐	For hierarchical and complex designs, identification of the appropriate level for tests and full reporting of outcomes
☐	☒	Estimates of effect sizes (e.g. Cohen's *d*, Pearson's *r*), indicating how they were calculated

Our web collection on statistics for biologists contains articles on many of the points above.

Software and code

Policy information about availability of computer code

Data collection	Stata (v16.1).
Data analysis	FastQC (v0.11.9), Trim Galore (v0.5.0), Cutadapt (v1.18), STAR (v2.5.4b), FastQC (v0.11.9), featureCounts (v2.0.1), DESeq2 (v1.30.1), heatmap.2 (v3.1.1), gProfiler (v2021-05-01), venn (v1.10), circlize (v0.4.1), MACS2 (v3.0.0a6), bowtie2 (v2.4.1), GREAT (v4.0.4), HOMER (v3.0), cellranger (v2.0.0), cicero (v1.18), R (v4.0.5), Stata (v16.1). References for all software packages and key parameters are listed in the Methods section.

For manuscripts utilizing custom algorithms or software that are central to the research but not yet described in published literature, software must be made available to editors and reviewers. We strongly encourage code deposition in a community repository (e.g. GitHub). See the Nature Portfolio guidelines for submitting code & software for further information.

Data

Policy information about availability of data

All manuscripts must include a data availability statement. This statement should provide the following information, where applicable:

- Accession codes, unique identifiers, or web links for publicly available datasets
- A description of any restrictions on data availability
- For clinical datasets or third party data, please ensure that the statement adheres to our policy

Cell line data were generated as described in the methods section. The raw bulk and single-cell RNA and ATAC sequencing data generated in this study have been deposited in the Gene Expression Omnibus (GEO) database under accession code GSE220144 [https://www.ncbi.nlm.nih.gov/geo/query/acc.cgi?acc=GSE220144].

The raw bulk and single-cell RNA and ATAC sequencing data generated in this study have been deposited in the Gene Expression Omnibus (GEO) database under accession code GSE220144 [https://www.ncbi.nlm.nih.gov/geo/query/acc.cgi?acc=GSE220144]. Publicly available datasets analyzed during the current study are available in Gene Expression Omnibus: GSE2658 (gene expression microarray data, reference 37) [https://www.ncbi.nlm.nih.gov/geo/query/acc.cgi?acc=GSE2658], GSE106218 (single-cell gene expression data, reference 61) [https://www.ncbi.nlm.nih.gov/geo/query/acc.cgi?acc=GSE106218], GSE24080 (gene expression microarray data, reference 77) [https://www.ncbi.nlm.nih.gov/geo/query/acc.cgi?acc=GSE24080], GSE117156 (single-cell gene expression data, reference 59) [https://www.ncbi.nlm.nih.gov/geo/query/acc.cgi?acc=GSE117156], and GSE110499 (single-cell gene expression data, reference 61) [https://www.ncbi.nlm.nih.gov/geo/query/acc.cgi?acc=GSE110499]. The MMRF IA16 bulk gene expression data was accessed through the MMRF Researcher Gateway (https://research.themmrf.org, reference 58).

The IFM dataset analyzed during the current study are available from the authors upon reasonable request. The remaining data are available within the Article file, Supplementary Information file, Supplementary Data files, or Source Data file.

Research involving human participants, their data, or biological material

Policy information about studies with human participants or human data. See also policy information about sex, gender (identity/presentation), and sexual orientation and race, ethnicity and racism.

Reporting on sex and gender	All analyses included both females and males. Please see the publications describing patient characteristics including sex distribution linked to the individual Gene Expression Omnibus data repositories: GSE2658, GSE106218, GSE24080, GSE117156, and GSE110499. Please see Attal et al. IFM 2009 Study. Lenalidomide, Bortezomib, and Dexamethasone with Transplantation for Myeloma. N Engl J Med 2017;376(14):1311-1320 for the IFM patient characteristics. The chosen multiple myeloma cell lines include both female and male karyotypes.
Reporting on race, ethnicity, or other socially relevant groupings	All analyses included patients with a diverse racial and ethnic background. Please see the publications describing patient characteristics including race and ethnicity distribution linked to the individual Gene Expression Omnibus data repositories: GSE2658, GSE106218, GSE24080, GSE117156, and GSE110499. Please see Attal et al. IFM 2009 Study. Lenalidomide, Bortezomib, and Dexamethasone with Transplantation for Myeloma. N Engl J Med 2017;376(14):1311-1320 for the IFM patient characteristics. The chosen multiple myeloma cell lines include both patients of African and European ancestry.
Population characteristics	Please see the publications describing patient characteristics linked to the individual Gene Expression Omnibus data repositories: GSE2658 / GSE24080 (age range 25-77 years, 40% women), GSE106218 / GSE110499 (age range 31-77 years, 53% women), GSE117156 (age range 40-84 years, 38% women). Please see Attal et al. IFM 2009 Study. Lenalidomide, Bortezomib, and Dexamethasone with Transplantation for Myeloma. N Engl J Med 2017;376(14):1311-1320 for the IFM patient characteristics (age range 29-66 years, 40% women). Please refer to the MMRF CoMMpass study whitepaper for information on the IA16 patient population (age range 27-93 years, 41% women) [https://www.themmrf.org/wp-content/uploads/MMRF-CoMMpass-Whitepaper.pdf]
Recruitment	Please see the publications describing patient characteristics linked to the individual Gene Expression Omnibus data repositories: GSE2658, GSE106218, GSE24080, GSE117156, and GSE110499. Please see Attal et al. IFM 2009 Study. Lenalidomide, Bortezomib, and Dexamethasone with Transplantation for Myeloma. N Engl J Med 2017;376(14):1311-1320 for the IFM recruitment strategy. Please refer to the MMRF CoMMpass study whitepaper for information on the IA16 patient population [https://www.themmrf.org/wp-content/uploads/MMRF-CoMMpass-Whitepaper.pdf]
Ethics oversight	Please see the publications describing patient characteristics linked to the individual Gene Expression Omnibus data repositories: GSE2658, GSE106218, GSE24080, GSE117156, and GSE110499. Please see Attal et al. IFM 2009 Study. Lenalidomide, Bortezomib, and Dexamethasone with Transplantation for Myeloma. N Engl J Med 2017;376(14):1311-1320 for the IFM ethics oversight. Please refer to the MMRF CoMMpass study whitepaper for information on the IA16 patient population [https://www.themmrf.org/wp-content/uploads/MMRF-CoMMpass-Whitepaper.pdf]. This research complies with all relevant ethical regulations of the participating institutions that approved the study protocol.

Note that full information on the approval of the study protocol must also be provided in the manuscript.

Field-specific reporting

Please select the one below that is the best fit for your research. If you are not sure, read the appropriate sections before making your selection.

☒ Life sciences ☐ Behavioural & social sciences ☐ Ecological, evolutionary & environmental sciences

For a reference copy of the document with all sections, see nature.com/documents/nr-reporting-summary-flat.pdf

Life sciences study design

All studies must disclose on these points even when the disclosure is negative.

Sample size	The sample size was chosen based on the RNA-seq power calculation (at least 80% power to detect a 3-fold or greater change in gene expression). The factors determining the required sample size (n) for this experimental design are the desired power (set as ≥ 80.0%, considered acceptable by the investigator), the α-level (set as 0.05, considered acceptable by the investigator), the effect size (ES, set as ≥ 3-fold change in gene expression for a given gene, considered biologically significant by the investigator), the average sequencing coverage (CO, set as ≥ 20, empirically derived from previous studies), and the coefficient of variation (CV, set as 0.4, empirically derived from a large number of human RNA-seq experiments, please see Hart et al. J Comput Biol 2013;20(12):970-978).
Data exclusions	For multivariable-adjusted regression modeling missing data was assumed to be missing completely at random (MCAR). The models therefore represent all patients with complete available data allowing for model fitting.
Replication	All RNA-seq and ATAC-seq experiments were performed in triplicate (biological replicates, n = 3). All attempts at replication were successful. All samples passed quality control and were included in the analysis.
Randomization	Sample characteristics were exchangeable at baseline by virtue of originating from isogenic cell lines. Therefore, randomization was not employed to allocate samples.
Blinding	Blinding was not possible at the experimental stage due to different experimental protocols for treatment and control groups. Blinding at the data analysis stage was not possible because knowledge of the sample group identity was required to carry out the comparisons of interest.

Reporting for specific materials, systems and methods

We require information from authors about some types of materials, experimental systems and methods used in many studies. Here, indicate whether each material, system or method listed is relevant to your study. If you are not sure if a list item applies to your research, read the appropriate section before selecting a response.

Materials & experimental systems

n/a	Involved in the study	
☒	☐	Antibodies
☐	☒	Eukaryotic cell lines
☒	☐	Palaeontology and archaeology
☒	☐	Animals and other organisms
☒	☐	Clinical data
☒	☐	Dual use research of concern
☒	☐	Plants

Methods

n/a	Involved in the study	
☒	☐	ChIP-seq
☒	☐	Flow cytometry
☒	☐	MRI-based neuroimaging

Eukaryotic cell lines

Policy information about cell lines and Sex and Gender in Research

Cell line source(s)	INA-6 (46, XY), male (Plasma Cell).<br>MM.1S (46, XX), female (Plasma Cell).<br>RPMI-8226 (46, XY), male (Plasma Cell).<br>HS-5 (46, XY), male (Bone Marrow Stroma Cell).
Authentication	The MM.1S, RPMI-8226, and HS-5 cells were obtained from ATCC and authenticated using ATCC STR profiling protocols (https://www.atcc.org/services/cell-authentication). INA-6 was obtained from DMSZ and authenticated using DMSZ STR profiling protocols (https://www.dsmz.de/collection/catalogue/human-and-animal-cell-lines/identity-control/authentication-of-cell-lines).
Mycoplasma contamination	All cell lines tested negative for Mycoplasma contamination.
Commonly misidentified lines<br>(See ICLAC register)	No commonly misidentified cell lines were used in the study.

Plants

Seed stocks	N/A
Novel plant genotypes	N/A
Authentication	N/A
